# Increasing the spectrum of white matter diseases with tigroid pattern on MRI: glutaric aciduria type 1 – case report

**DOI:** 10.1186/s12887-021-02603-5

**Published:** 2021-03-27

**Authors:** Monika Bekiesinska-Figatowska, Marek Duczkowski, Agnieszka Duczkowska, Joanna Taybert, Amanda Krzywdzinska, Jolanta Sykut-Cegielska

**Affiliations:** 1 Department of Diagnostic Imaging, Institute of Mother and Child, Kasprzaka 17a, 01-211 Warsaw, Poland; 2 Department of Inborn Errors of Metabolism and Paediatrics, Institute of Mother and Child, Warsaw, Poland

**Keywords:** Glutaric aciduria type I (GA-1), Magnetic resonance imaging (MRI), White matter, Tigroid pattern, Forniceal injury, Memory impairment, Child, Case report

## Abstract

**Background:**

Most white matter diseases present on magnetic resonance imaging as focal or diffuse T2-hyperintensities. However, in a few of them, radially oriented stripes of low (relatively normal) signal intensity are observed within diffusely affected T2-hyperintense cerebral white matter and are called “tigroid pattern” in the literature. The fornix is a tiny white matter fibers bundle playing crucial role in cognitive functioning, easily overlooked on magnetic resonance imaging and not described in inborn errors of metabolism.

**Case presentation:**

We present a case of glutaric aciduria type 1 with a follow-up of over nine years. The course of the disease is presented in three magnetic resonance scans at the age of 8 and 21 months, and 10 years, with diffusion restriction in the fornix in scan 1 and 2 and with tigroid pattern in scan 3. Despite appropriate diet and supplementation, injury of white matter progressed achieving diffuse stage with tigroid pattern. Psychological tests revealed deficits in patient’s specific cognitive skills, most likely related to damage to the fornix.

**Conclusions:**

To our knowledge, this is the first report of tigroid pattern of white matter involvement in glutaric aciduria type 1 and the first report of forniceal injury in this disease which seems to be correlated with patient’s low functioning in all kinds of memory skills, previously not reported in glutaric aciduria type 1.

## Background

Most white matter (WM) diseases present on magnetic resonance imaging (MRI) as focal or diffuse fluid-attenuated inversion recovery (FLAIR)- and T2-hyperintensities. However, in some of them, the radially oriented stripes of low (relatively normal) signal intensity (SI) are observed within diffusely affected T2-hyperintense cerebral WM and are called “tigroid pattern” or “leopard skin pattern” in the literature. Tigroid pattern was first described in Pelizaeus-Merzbacher disease (PMD) and metachromatic leukodystrophy (MLD) [1] and later on in a few other inborn errors of metabolism: Lowe syndrome, Alexander disease, as well as in an acquired myelin disease: acute disseminated encephalomyelitis (ADEM) [2–6]. Neuropathological studies in some lysosomal storage disorders elucidated the reason of such involvement and confirmed the radiological hypothesis that the stripes of apparently unchanged SI correspond to relative sparing of myelin in perivenular regions in MLD, and infantile GM1 gangliosidosis (GM1); in MLD there were lipid-containing glial cells in these areas as well. Interestingly, in Krabbe disease (GLD) no myelin was found in these areas and only lipid-containing globoid cells corresponded to the stripes [7].

In recent years, the fornix has been in focus of researchers working on understanding the mechanisms and finding effective prevention and treatment of Alzheimer’s disease. However, any kind of neurodegenerative, vascular or traumatic lesions of the fornix, as a part of the Papez circuit, can cause memory deficits [8] so the metabolic injury must also be taken into account. Being a relatively small WM fibers bundle, the fornix is easily overlooked on MRI.

We present a case of glutaric aciduria type 1 (GA-1) with tigroid pattern of WM involvement and forniceal involvement, together with its possible impact on patient’s specific cognitive skills.

## Case presentation

The boy was born at 42 gestational weeks by Caesarean section due to lack of delivery progress with body length of 55 cm, body weight of 3 720 g, head circumference of 38 cm, and Apgar score of 8/10 points (at 1st/5th minute). Because of marked increase of head circumference and slight developmental delay, after 6 months brain ultrasound was performed (which was normal) and physiotherapy was started. At the age of 8 months he slipped off the couch and underwent head injury. Computed tomography (CT) was performed and did not reveal trauma-related abnormalities. Due to other abnormal findings, misinterpreted as Dandy-Walker malformation suspicion, MRI was then performed (scan 1), also outside our center, and this time Wilson disease was suspected but not confirmed by biochemical or DNA tests. At the age of 9 months the boy was unable to sit down by himself. At the age of 17 months he underwent extensive neurologic examination and metabolic work-up based on urinary organic acid GC/MS profile which revealed: massive (1374 mmol/mol creatinine) excretion of glutaric acid, significantly increased (79 mmol/mol creatinine) 3-hydroxyglutaric acid and small glutarylglycine, consistent with glutaric aciduria type 1. Acylcarnitine profile in dry blood spot showed the following findings: C5DC/C8i – 0.53 umol/L (normal value (N) < 0.3 umol/L), free carnitine – 5.9 umol/L (N 7.3–86 umol/L) and total carnitine − 15.7 umol/L (N 17–134 umol/L). The diagnosis of GA-1 was confirmed by DNA analysis, which identified the known pathogenic variant c.1204 C > T (p.Arg402Trp) in GCDH gene in homozygous status. The family history was unremarkable. The somatic parameters were as follows: the patient’s weight 15.5 kg (90 percentile), height 98 cm (> 97 percentile), head circumference 52 cm (> 97 percentile).

Since diagnosis the boy has been under the care of metabolic center, his treatment includes low-lysine and low-tryptophan diet, carnitine supplementation and emergency management. His dietary restriction was slightly relaxed after 6 years of age, but the boy is still under care of a dietitian.

The patient is now 10 years old. No clinically evident metabolic decompensations have occurred until now. Neither dystonic nor other movement disorders have been observed up to now. His development seems to be normal, but with low functioning in some specific psychological domains. According to his mother’s report, learning difficulties are the boy’s main problem now.

The patient earned Full Scale Intelligence Quotient (FSIQ) score of 95 on Stanford-Binet Intelligence Scales, Fifth Edition. His overall intelligence is classified as average (34th percentile). There is a 90 % probability that his ‘true’ FSIQ is 90–100. We observe discrepancies among the specific domains. Working memory represents patient’s poorest area of performance. He has diminished ability to acquire and store various information in short-term memory, to “transform” or “sort” this information, and to analyze it. Working memory is proven to be of great importance in school learning and general problem solving. Compared to other individuals, the patient’s standard score of 80 would be described as Low Average. Patient underwent additional assessment of particular cognitive, psycho-motoric, language, social and emotional functions with Intelligence and Development Scales (IDS) due to poor results of tests assessing working memory, difficulties in complex verbal messages comprehension, and difficulties in school learning and motor processes reported by his mother. The results are presented in Table 1.

**Table 1:** Intelligence and Development Scales (IDS) results of our patient

Test	Sub-tests	Raw score	Standard score (confidence interval 85 %)	Result
**Cognitive skills**	Visual perception	31	10 (12) 14	average
	Selective attention	32	3 (4) 5	low
	Phonological memory	5	4 (6) 8	low
	Visual-spatial memory	22	5 (6) 7	low
	Spatial reasoning	7	10 (12) 14	average
	Conceptual reasoning	4	2 (4) 6	low
	Auditory memory	25	4 (5) 6	low
**Psycho-motor skills**	Motorics	6	4 (6) 8	low
	Manipulation	6	0 (3) 6	low
	Visual and auditory co-ordination	11	8 (11) 14	average
**Social and emotional competence**	Emotion recognition	9	10 (12) 14	average
	Emotion regulation	13	8 (10) 12	average
	Social understanding	9	4 (7) 10	low average
	Social behavioural competence	10	8 (10) 12	average
**Mathematical skills**	Logical and mathematical reasoning	12	8 (9) 10	average
**Language skills**	Active speech	10	8 (10) 12	average
	Passive speech	7	4 (5) 6	low

**Fig. 1:**
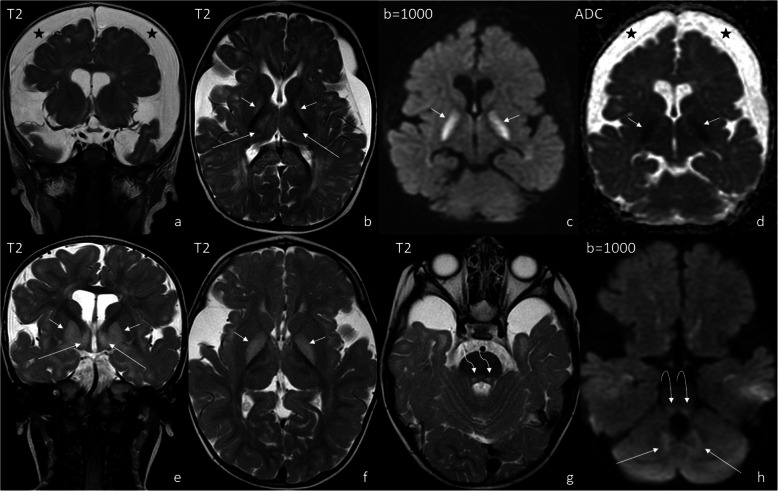
Typical MRI features of GA-1. Scan (1) at the age of 8 months - upper row, scan (2) at the age of 21 months – lower row. Abnormal T2-hyperintensity and diffusion restriction of the globi pallidi in both examinations (b-d and e,f, short arrows). Dentate nuclei (**h**) and thalami (**b**,**e**) with T2-hyperintensity and diffusion restriction in both examinations (long arrows). Medial lemnisci in dorsal pons with T2-hyperintensity and diffusion restriction in both examinations (**g**,**h**, curved arrows). Symmetrical subdural hygromas (asterisks) detected on scan (1) (**a**-**d**) regressed in scan (2) (**e**-**h**)

**Fig. 2:**
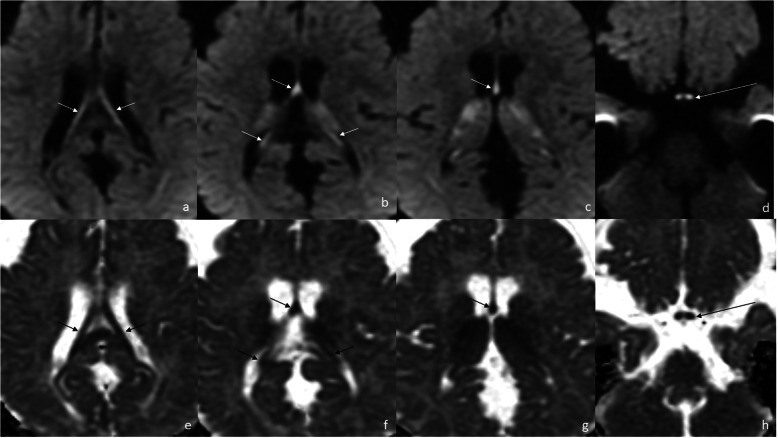
Scan 1) at the age of 8 months. Diffusion restriction in the fornix (**a**-**c**, **e**-**g**, short arrows) and in the optic chiasm (**d**, **h**) (**a**-**d**: DWI, **e**-**h**: ADC maps, long arrows). The involvement of the globi pallidi is also seen (**c**, **g**)

**Fig. 3:**
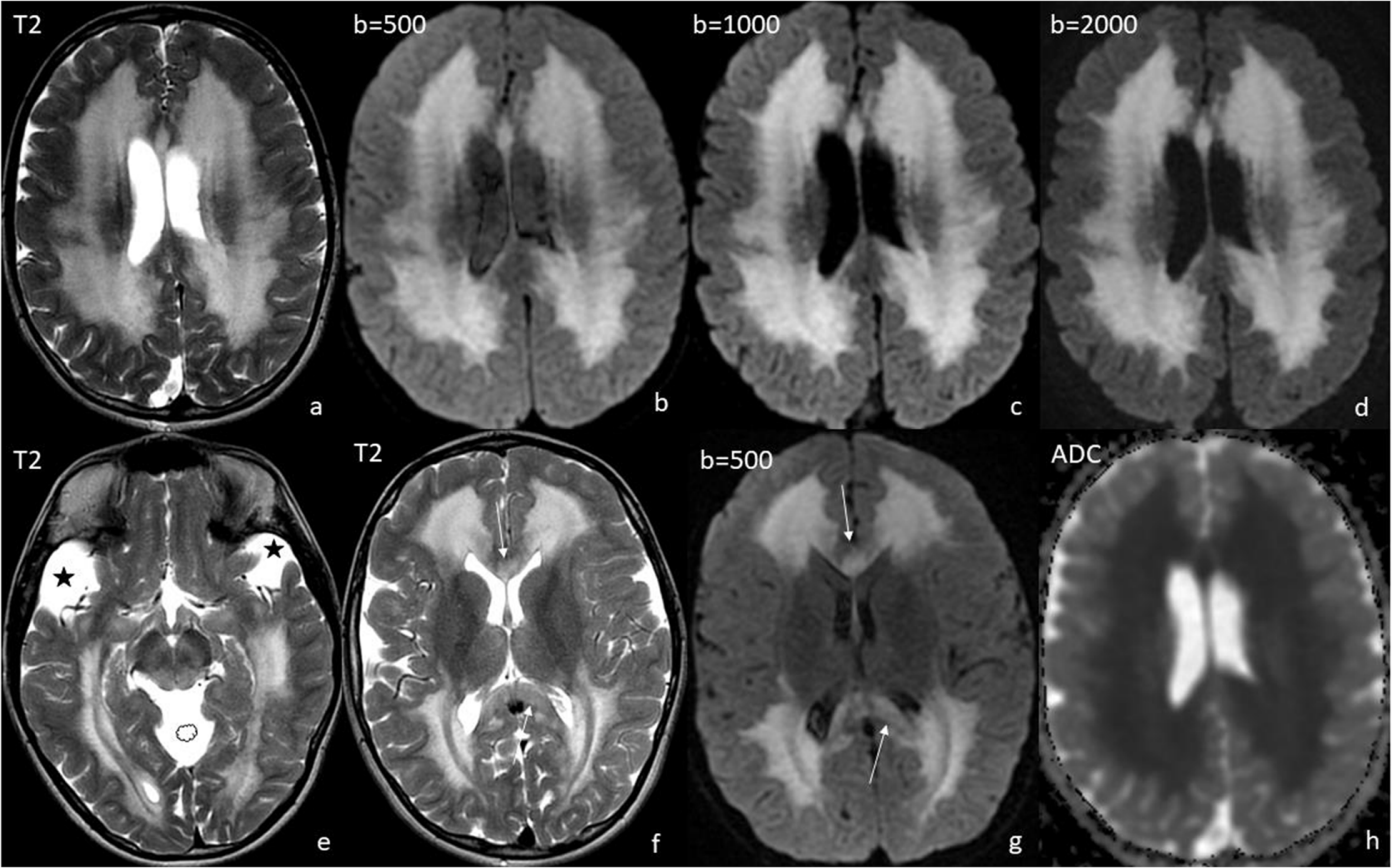
Scan 3) at the age of 10 years. Diffuse symmetrical white matter involvement with tigroid pattern (**a**-**g**) and diffusion restriction (**h**). Note regression of subdural hygromas and of lesions in the pallidum as well as callosal involvement (**f**, **g**, arrows). Widened Sylvian fissures (asterisks) and mesencephalic cistern (cloud) as a constant feature of GA-1 (**e**)

**Fig. 4:**
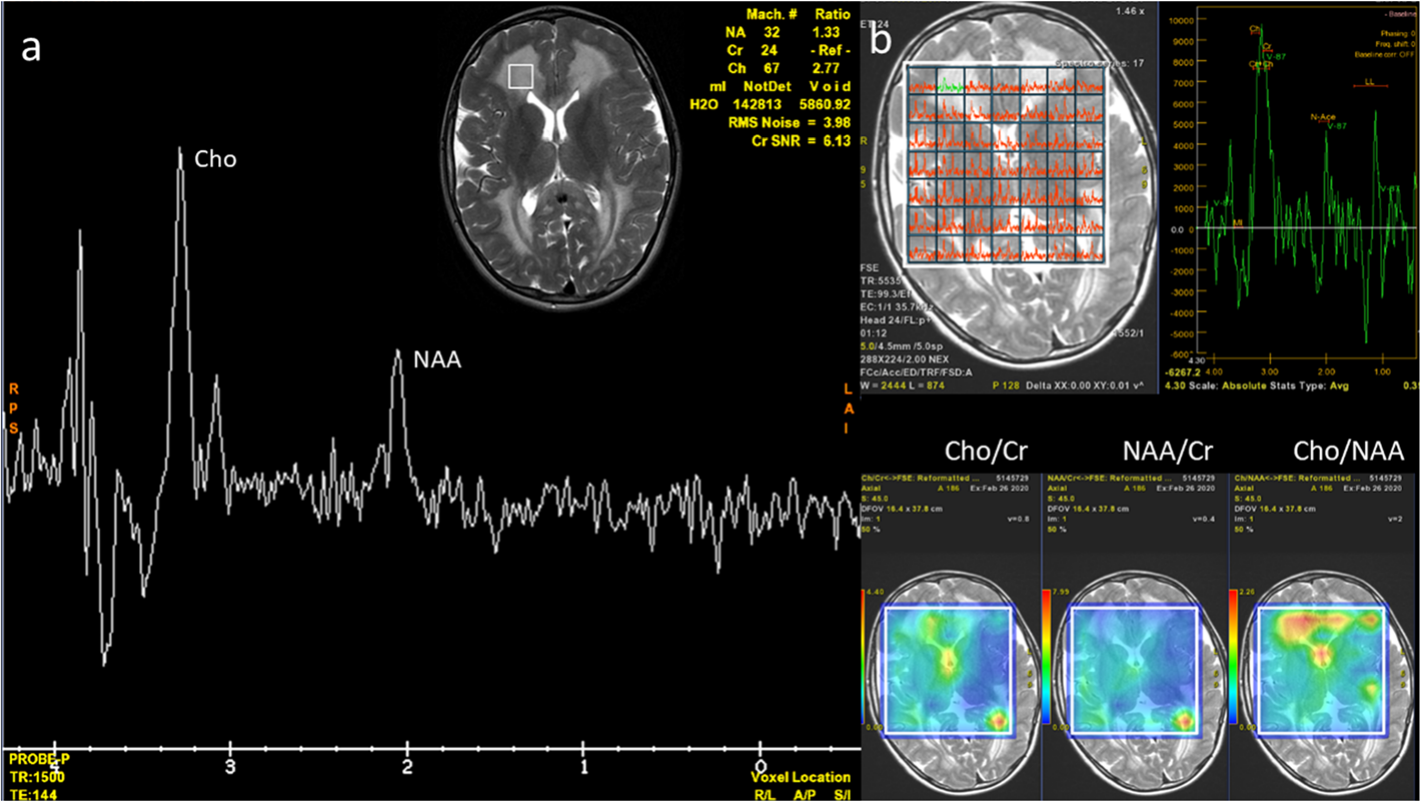
MR spectroscopy at the age of 10 years. Both single-voxel (**a**) and multivoxel (**b**) MRS show decrease of N-acetylaspartate (NAA) and increase of choline (Cho)

Three MRI scans were performed and analyzed: (1) at the age of 8 months, (2) – 21 months, (3) – 10 years. They were all performed with use of 1.5T GE scanners, the first two SIGNA HDxt, the third – SIGNA Artist. T1-, T2-weighted, T2flair, SWI and DWI images were obtained in three planes (axial, coronal and sagittal). Striatum was not affected in our patient. Abnormal T2-hyperintensity and diffusion restriction were present in the globi pallidi in scan (1) and (2) (Fig. 1b-f) and subsided in scan 3). Dentate nuclei (Fig. 1 h) and thalami (Fig. 1b,e) showed T2-hyperintensity on all scans, with diffusion restriction in scan 1) and 2). The fornix, optic chiasm (Fig. 2) and medial lemnisci in dorsal pons (Fig. 1 g,h) displayed high SI on T2-weighted images and diffusion restriction on scan 1) and 2), this resolved in scan 3). Myelination was delayed in scan 1) and progressed in scan 2). Scan 3) showed diffuse bilateral symmetrical leukoencephalopathy with callosal involvement and diffusion restriction. The pattern of hemispheric WM involvement, with stripes and dots of relatively normal SI within diffuse abnormalities was consistent with tigroid pattern described in other diseases but not in GA-1 (Fig. 3). MR spectroscopy (MRS) was performed on scan 3). Both single- and multivoxel techniques were applied. In single-voxel MRS the voxels were placed in the tigroid pattern areas in the frontal lobes. MRS showed decreased N-acetylaspartate (marker of neuronal density) at 2.0 ppm, and increased choline (marker of the synthesis and breakdown of cell membranes) at 3.2 ppm (Fig. 4). Symmetrical subdural hygromas detected on scan (1) (Fig. 1a-d) regressed in scan (2) (Fig. 1e-h) and 3). Width of the Sylvian fissures decreased with time. Prominent frontal horns of the lateral ventricles (Fig. 1a,e), widened mesencephalic cistern (Fig. 3e) and anterior temporal subarachnoid spaces (Fig. 1 g) were constant features.

## Discussion and conclusion

Glutaric aciduria type 1 (Online Mendelian Inheritance in Man, OMIM: 231,670) is an autosomal recessive inborn error of metabolism caused by deficiency of glutaryl-CoA dehydrogenase, which leads to accumulation of 3-hydroxyglutaric and glutaric acid in brain which, in turn, causes striatal injury and myelinopathy [9]. The disease occurs in approximately 1 in 100,000 individuals, and is more common in the Amish community and in the Ojibwa population of Canada, where it occurs up to 1 in 300 newborns.

The general MRI picture of GA-1 in our case, as described above, is in accordance with previously reported features [10–12]. The MRI pattern of brain involvement with fluid collections over the convexities, wide open Sylvian fissures and affected basal ganglia, substantia nigra, dentate nuclei has been described for years as highly suggestive of glutaric aciduria type 1 [13]. Our GA-1 patients from the earlier studies showed involvement of the lentiform nuclei and variable degree of WM involvement: from no visible changes in a 5-year-old boy through hypomyelination only (e.g. in a 16-day term neonate; this baby – as the only GA-1 patient - did not show involvement of deep grey matter structures) to patchy, asymmetrical T2-hyperintensities. All of them shared the picture of widely open Sylvian fissures. Our present patient belongs to the group of GA-1 patients without striatal lesions on MRI, and with affected globi pallidi, thalami and dentate nuclei. Confluent WM changes in GA-1 are known as well as restricted (or elevated) diffusion [11], however, the presumed sparing of perivenular myelin found in our case and presenting as “tigroid pattern” has not been published yet. The assessment of the causal relationship between tigroid appearance of WM on MRI and glutaric aciduria type 1 is beyond the scope of our research capabilities as radiologists and specialists in pediatric metabolic medicine. Even the pathological results in cases of autopsies in three different diseases with tigroid pattern on MRI (although all three belonging to the group of lysosomal storage disorders: MLD, GLD and GM1) have shown that different histopathologic correlates are responsible for a similar characteristic MRI pattern [7]. In very few publications devoted to neuropathology of GA-1, mainly pathophysiology of striatal degeneration is in focus, however, it has been found that WM abnormalities on MRI reflect spongiform myelinopathy which seems to progress with age [11]. This progression is extensive and obvious in our case in scan 3) despite treatment and it should be clearly stated that diffusion restriction does not reflect an acute encephalopathic crisis in our patient who was in stable good clinical condition before, during and after scan 3). This is in contrast with some of the published cases that link diffusion restriction to metabolic crisis [14]. There are not many reports concerning serial scanning of GA-1 patients over the long periods of time; in one of them 2/10 patients had no change in the MRI appearance over time, 1/10 showed slight worsening, and 7/10 - variable degree of improvement in the imaging findings [10]. In a richly illustrated paper by Harting et al. analyzing brain MRI in 38 GA-1 patients we found one case of similarly extensive WM changes in a 9.5-year-old boy examined 1 year after the diagnosis. These authors state that the evolving T2 WM signal abnormalities are typical of late-onset disease and that neurotoxic dicarboxylic acids interfere with gyration and opercularization as well as with maturation of WM (manifesting as myelination delay) and maintenance of WM (manifesting as its T2-hyperintensity) [11].

We also noticed diffusion restriction in the optic chiasm that was mentioned once in the literature [15] and previously undescribed involvement of the fornix with diffusion restriction. Ocular abnormalities in GA-1 patients, such as intraretinal hemorrhage, cataract, gaze palsy, strabismus, ametropia, and pigmentary retinopathy have been described in the literature [16]. Serrano Russi et al. have only pointed at diffusion restriction in the optic chiasm in one of their patients but did not describe any further consequences of it to this child that has been followed-up for the next 3.5 years [15]. In our patient the significance of this finding after 9 years is still unknown.

Of the metabolic diseases, Wernicke encephalopathy is the (acquired) one in which the forniceal finding was reported [17]. The fornix is a part of the limbic system, connects the hippocampus to the hypothalamus and as a component of Papez circuit plays an important role in formation of memory. Abnormalities within the fornix may contribute to memory impairment/cognitive dysfunction. This has been shown in mild cognitive impairment and Alzheimer’s disease, multiple sclerosis, schizophrenia [17]. Damage to the fornix has not been reported in association with GA-1 so far – this is the first case - and cognitive function seems to be preserved in these patients [18]. Boy et al. were the first to investigate as many as 30 patients with confirmed diagnosis of GA-1 in order to analyze their neuropsychological functions (in general, the biggest described group of GA-1 patients that we found in the literature included 77 individuals [19]). They also reviewed the literature for cognitive research and stated that memory deficits had not been reported in GA-1 patients – except for 2 cases [18].

We were able to show that forniceal changes correlate with our patient’s low functioning in specific cognitive skills such as all kinds of memory skills, as well as selective attention, gross and fine motor skills and passive speech.

To our knowledge this is the first description of tigroid pattern of white matter involvement in glutaric aciduria type 1. The affected fornix has not been described so far either in inborns errors of metabolism in general and in GA-1 in particular. This report expands the spectrum of clinical and MRI features in GA-1 and the spectrum of diseases with tigroid pattern of WM involvement.

Our report also emphasizes the importance of consulting MR examinations at dedicated referral centers in order to shorten the time to diagnosis and to implementation of appropriate treatment.

## Data Availability

The data used to support the findings of this study are available from the corresponding author upon request.

## Abbreviations

WM: White matter
MRI: Magnetic resonance imaging
FLAIR: Fluid-attenuated inversion recovery
SI: Signal intensity
PMD: Pelizaeus-Merzbacher disease
MLD: Metachromatic leukodystrophy
ADEM: Acute disseminated encephalomyelitis
GM1: GM1 gangliosidosis
GLD: Globoid-cell leukodystrophy, Krabbe disease
FSIQ: Full Scale Intelligence Quotient
IDS: Intelligence and Development Scales
MRS: Magnetic resonance spectroscopy
ppm: Parts per million
OMIM: Online Mendelian Inheritance in Man
DWI: Diffusion-weighted imaging
ADC: Apparent diffusion coefficient
NAA: N-acetyloaspartate
Cho: Choline
